# Citrullination-Resistant LL-37 Is a Potent Antimicrobial Agent in the Inflammatory Environment High in Arginine Deiminase Activity

**DOI:** 10.3390/ijms21239126

**Published:** 2020-11-30

**Authors:** Danuta Bryzek, Anna Golda, Joanna Budziaszek, Dominik Kowalczyk, Alicia Wong, Ewa Bielecka, Priyanka Shakamuri, Pavel Svoboda, Jan Pohl, Jan Potempa, Joanna Koziel

**Affiliations:** 1 Department of Microbiology, Faculty of Biochemistry, Biophysics and Biotechnology of Jagiellonian University, 30-387 Krakow, Poland; danuta.bryzek@uj.edu.pl (D.B.); anna.b.golda@uj.edu.pl (A.G.); joanna.budziaszek@doctoral.uj.edu.pl (J.B.); dominik.kowalczyk@doctoral.uj.edu.pl (D.K.); aliciawong.ky@gmail.com (A.W.); 2 Malopolska Center of Biotechnology, Jagiellonian University, 30-387 Krakow, Poland; ewa.bielecka@uj.edu.pl; 3 Biotechnology Core Facility Branch, Division of Scientific Resources, Centers for Disease Control and Prevention, Atlanta, GA 30333, USA; wpd5@cdc.gov (P.S.); hug6@cdc.gov (P.S.); hhe7@cdc.gov (J.P.); 4 Department of Oral Immunity and Infectious Diseases, University of Louisville School of Dentistry, University of Louisville, Louisville, KY 40202, USA

**Keywords:** LL-37, arginine, homoarginine, citrullination, NETs

## Abstract

LL-37, the only member of the mammalian cathelicidin in humans, plays an essential role in innate immunity by killing pathogens and regulating the inflammatory response. However, at an inflammatory focus, arginine residues in LL-37 can be converted to citrulline via a reaction catalyzed by peptidyl-arginine deiminases (PAD2 and PAD4), which are expressed in neutrophils and are highly active during the formation of neutrophil extracellular traps (NETs). Citrullination impairs the bactericidal activity of LL-37 and abrogates its immunomodulatory functions. Therefore, we hypothesized that citrullination-resistant LL-37 variants would retain the functionality of the native peptide in the presence of PADs. To test this hypothesis, we synthetized LL-37 in which arginine residues were substituted by homoarginine (hArg-LL-37). Bactericidal activity of hArg-LL-37 was comparable with that of native LL-37, but neither treatment with PAD4 nor exposure to NETs affected the antibacterial and immunomodulatory activities of hArg-LL-37. Importantly, the susceptibilities of LL-37 and hArg-LL-37 to degradation by proteases did not significantly differ. Collectively, we demonstrated that citrullination-resistant hArg-LL-37 is an attractive lead compound for the generation of new agents to treat bacterial infections and other inflammatory diseases associated with enhanced PAD activity. Moreover, our results provide a proof-of-concept for synthesis of therapeutic peptides using homoarginine.

## 1. Introduction

Antimicrobial peptides (AMPs) have a broad spectrum of antibacterial and immunomodulatory activities and constitute an ancient branch of the innate immune system [1,2]. The cathelicidin family of proteins was identified in mammals, with LL-37 being the only member in humans [3]. LL-37 is released from an 18-kDa precursor protein (hCAP-18) by protease-3 in neutrophils [4] and by a serine protease belonging to the kallikrein family in keratinocytes [5] and contains 37 amino acids with two leucine residues at its N-terminus. This cationic and amphipathic peptide kills microbes by binding to and permeabilizing the negatively charged bacterial cell membrane [6,7]. In addition, LL-37 reduces the interaction of endotoxin with LPS-binding protein and the TLR4 receptor and thus quenches the proinflammatory response of macrophages induced by LPS [8]. This is crucial to protect against sepsis during infections of Gram-negative bacteria [9]. At an inflammatory focus, LL-37 is exposed to calcium-dependent peptidyl-arginine deiminases (PADs), mainly PAD2 and PAD4 released from phagocytes, which convert arginine residues into citrulline [10]. Citrullinated LL-37 has been detected in inflamed lungs and neutrophil extracellular traps (NETs) in vivo [11,12]. Citrullination substantially decreases the positive charge of LL-37 and thereby compromises its biological functions [13]. Citrullinated LL-37 exhibits impaired antibacterial activity against *Escherichia coli*, a diminished ability to neutralize LPS, and reduced binding to nucleic acids [11,12,14]. Therefore, we postulate that prevention of LL-37 citrullination is a promising therapeutic strategy. To test this hypothesis, we synthesized LL-37 in which arginine residues were substituted by citrullination-insensitive homoarginine (hArg-LL-37) and compared the biological activities of hArg-LL-37 and the native peptide. Our findings indicate that hArg-LL-37 can be used as a therapeutic replacement for LL-37 in pathological inflammatory conditions such as psoriasis and rheumatoid arthritis, in which citrullination of the native peptide compromises its antibacterial and immunoregulatory activities [15,16].

## 2. Results

### 2.1. Antibacterial Activities of Native LL-37 and hArg-LL-37

**Figure 1:**
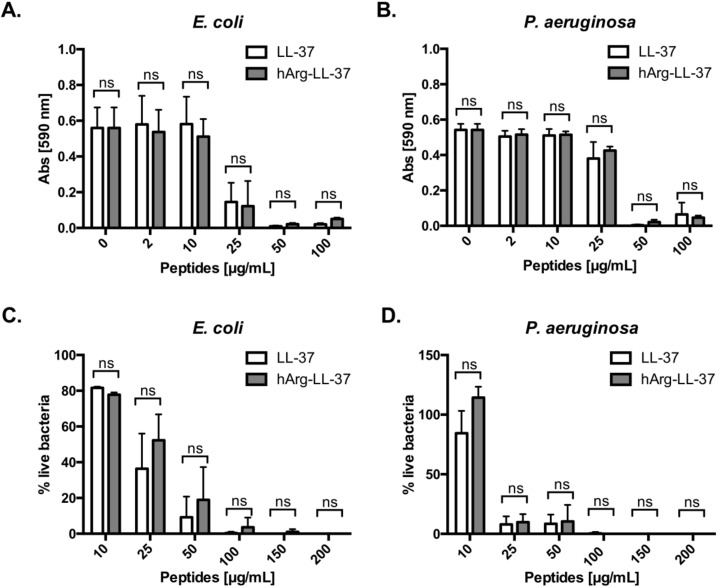
Antibacterial properties of LL-37 (native peptide) and hArg-LL-37 (synthetic peptide in which arginine residues were substituted for by citrullination-resistant homoarginine residues). Antimicrobial activity of LL-37 and hArg-LL-37 was assessed using a microdilution method as a standard protocol. The MIC (minimal inhibitory concentration) of tested peptides against Gram-negative (**A**) *E. coli* and (**B**) *P. aeruginosa*. Mean ± SD *n* = 2. ns—non-significant; Bactericidal effect of LL-37 and hArg-LL-37 on (**C**) *E. coli* and (**D**) *P. aeruginosa* were determined using SYTO9 and PI staining. Bacteria were treated with peptides (0–200 μg/mL) for 2 h and stained with SYTO9 and PI. The fluorescence was measured (excitation at 485 nm and emission at 530 nm (SYTO9) and 630 nm (PI)). Mean ± SD *n* = 3; ns—non-significant.

To examine the antibacterial activities of LL-37 and its analog hArg-LL-37 against Gram-positive and Gram-negative bacteria, we determined their minimum inhibitory concentrations. Of note, we did not investigate citrullinated forms of LL-37, as their impaired bacteriostatic and bactericidal activity in comparison to native LL-37 has already been comprehensively characterized [11,13]. Treatment with 50 μg/mL LL-37 or hArg-LL-37 completely inhibited growth of *E. coli* and *Pseudomonas aeruginosa* (MIC = 50 μg/mL) (Figure 1A,B). Next, we examined the bactericidal activities of the peptides using LIVE/DEAD staining. Treatment with either peptide at a concentration of 100 μg/mL effectively killed both bacterial species (Figure 1C,D). Taken together, these results indicate that the bacteriostatic and bactericidal activities of native LL-37 do not differ from those of hArg-LL-37.

### 2.2. Immunomodulatory Activities of Native LL-37 and hArg-LL-37

**Figure 2:**
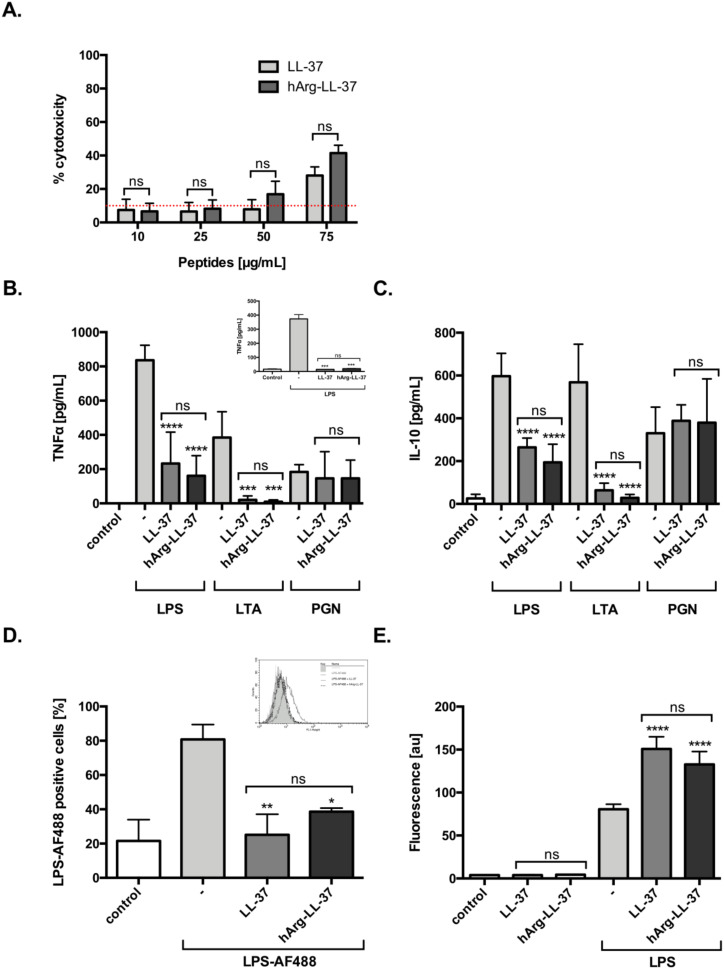
Immunomodulatory activities of LL-37 and hArg-LL-37. (**A**) The toxicity of LL-37 and hArg-LL-37 on HaCaT cells was evaluated using the LDH assay. Cells were plated on 96-well plates and incubated overnight. Next, macrophages were treated with the peptides at different concentrations (10–75 μg/mL) for 24 h. Mean ± SD *n* = 2. ns—non-significant. Human MDMs were stimulated with 10 ng/mL LPS, 10 μg/mL LTA and 10 μg/mL PGN in the presence of peptides (10 μg/mL). The level of (**B**) TNF-α and (**C**) IL-10 in the culture supernatants was measured by using ELISA at 20 h post-stimulation. Mean ± SD *n* = 3; ns—non-significant, *** *p* < 0.001, **** *p* < 0.0001. (**B-insert**) Human blood was treated with 10 ng/mL LPS in the presence of each peptide (10 μg/mL) and TNF-α concentration in plasma was measured 20 h later. Mean ± SD *n* = 2; ns—non-significant, *** *p* < 0.001; (**D**) The tested peptides blocked binding of AF488-conjugated LPS (AF488-LPS) to RAW 264.7 cells. Mouse macrophages (5 × 10^5^ cells/mL) were incubated for 15 min at 37 °C with 100 ng/mL AF488-LPS in the absence or presence of LL-37 and hArg-LL-37 (25 μg/mL). After washing, binding of AF488-LPS was analyzed by using flow cytometry. Background fluorescence was determined using RAW 264.7 cells incubated in the absence of AF488-LPS. A representative histogram from one of three independent experiments is shown, along with the mean percentage of macrophages that bound LPS (**D-insert**). Mean ± SD *n* = 3; ns—non-significant, * *p* < 0.05, ** *p* < 0.01; (**E**) ThT assay identified the amyloid formation by LL-37 and hArg-LL-37 (100 μg/mL) after addition of 100 μg/mL LPS. Mean ± SD *n* = 2; ns—non-significant, **** *p* < 0.0001.

LL-37 exerts a variety of immunomodulatory effects on different types of host cells, including neutralization of the proinflammatory potential of LPS and lipoteichoic acid (LTA) [14]. Therefore, we examined the anti-inflammatory functions of LL-37 and hArg-LL-37. First, we evaluated the dose-dependent toxicity of LL-37 and hArg-LL-37 against human keratinocytes using the LDH assay. We found that both tested peptides at 10 and 25 μg/mL did not compromise the membrane permeability of HaCaT cells. LL-37 and hArg-LL-37 triggered a low, but still significant, release of LDH at 75 μg/mL (Figure 2A). Human macrophages were stimulated with various pathogen-associated molecular patterns (LPS, LTA, and peptidoglycan (PGN)) in the presence or absence of LL-37 or hArg-LL-37 at concentrations that are not toxic to eukaryotic cells (10 μg/mL). LL-37 and hArg-LL-37 decreased LPS- and LTA-induced release of tumor necrosis factor (TNF)-α and interleukin (IL)-10 by macrophages with a similar efficiency (Figure 2B,C). Furthermore, to mimic physiological conditions, we examined the immunomodulatory properties of LL-37 and hArg-LL-37 against LPS in peripheral human blood and found no difference in their abilities to reduce the level of TNF-α (Figure 2B; insert). Moreover, we used fluorescently labeled LPS (LPS-AF488) to examine the abilities of LL-37 and hArg-LL-37 to interfere with the interaction of LPS with the cell membrane of macrophages. Flow cytometric analysis demonstrated that only 40% of cells were LPS-positive, indicating that both LL-37 and hArg-LL-37 limited binding of LPS to the cell membrane (Figure 2D). AMPs form aggregates with LPS, which are organized into amyloid-like structures. Therefore, we used the ThT fluorescent dye, which is widely used to selectively monitor amyloid formation and preferentially binds to β-sheet structures of amyloidogenic proteins and peptides. Significant binding of ThT was observed when LPS was exposed to LL-37 and hArg-LL-37, although aggregation increased to a similar extent (Figure 2E). Taken together, these data clearly demonstrate that hArg-LL-37 is as potent an anti-inflammatory agent as native LL-37.

### 2.3. Sensitivities of Native LL-37 and hArg-LL-37 to Proteolysis

**Figure 3:**
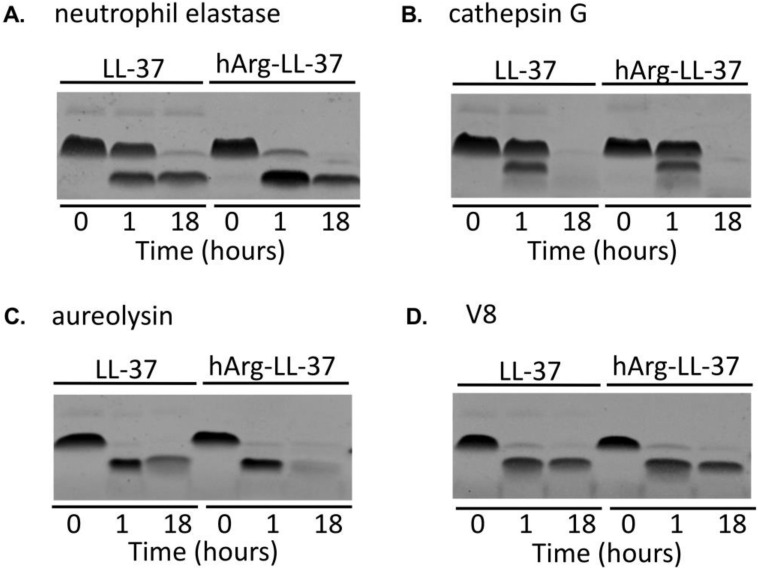
Degradation of native LL-37 and hArg-LL-37 by human and bacterial proteases. Both peptides were incubated with (**A**) neutrophil elastase; (**B**) cathepsin G; (**C**) aureolysin; and (**D**) V8 proteases for 1 or 18 h. Afterwards, the peptides were separated by SDS-PAGE and visualized using Coomassie staining.

Enhanced proteolytic activity is a hallmark of inflammation [17]. Given that hArg-LL-37 is a potential therapeutic peptide, it was necessary to determine its susceptibility to degradation by proteases. We compared the stabilities of LL-37 and hArg-LL-37 in the presence of host and bacterial proteolytic enzymes. LL-37 and hArg-LL-37 were incubated with two neutrophil-derived proteases, namely, neutrophil elastase and cathepsin G, for 1 and 18 h (Figure 3A,B). SDS-PAGE analysis showed that the susceptibilities of LL-37 and hArg-LL-37 to be degraded by cathepsin G did not differ (Figure 3B). However, hArg-LL-37 was degraded more rapidly by neutrophil elastase than LL-37 (Figure 3A). Conversely, the two peptides were similarly sensitive to proteolytic degradation by aureolysin and V8 protease (Figure 3C,D). Taken together, the sensitivities of LL-37 and hArg-LL-37 to proteolytic degradation did not differ in general, with the exception that hArg-LL-37 was more prone to cleavage by neutrophil elastase than the native peptide.

### 2.4. Antibacterial Properties of hArg-LL-37 in the Presence of Active PAD4

**Figure 4:**
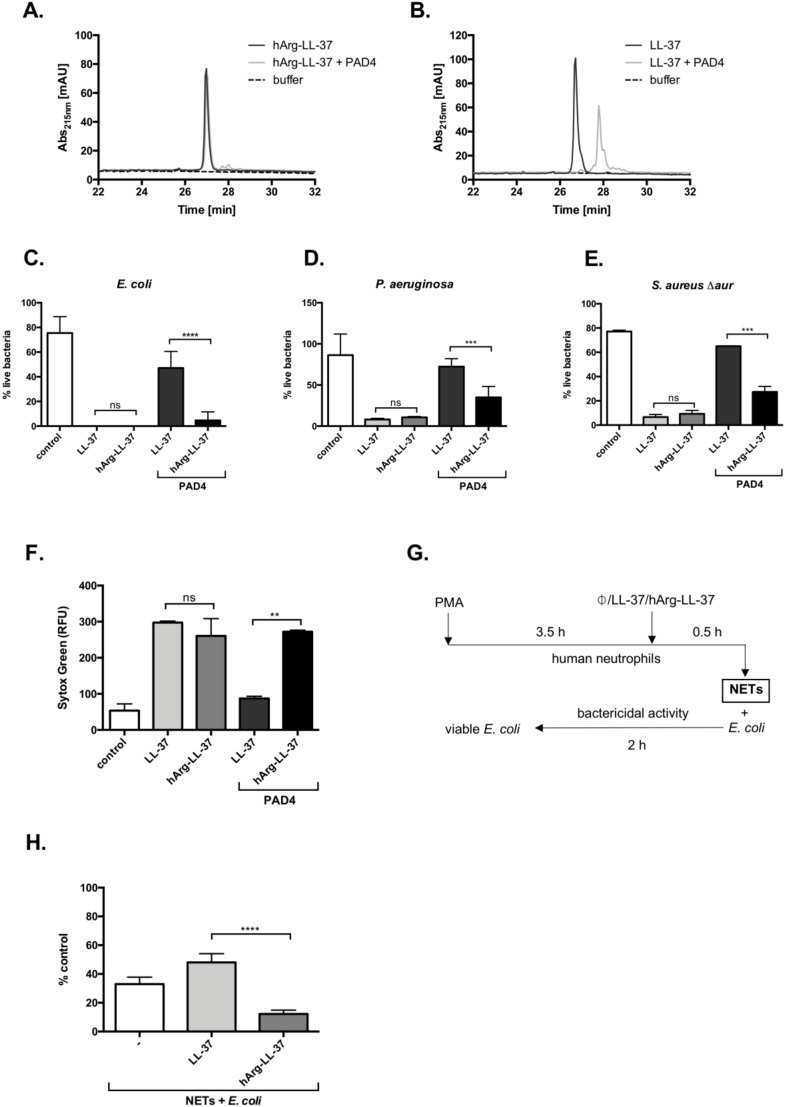
Citrullination abolishes the antimicrobial activity of native LL-37 but not hArg-LL-37. HPLC analysis of (**A**) hArg-LL-37 and (**B**) native LL-37. Samples containing 10 μg of LL-37 or hArg-LL-37 were treated with 233 mU of recombinant PAD4-HisTag for 2 h at 37 °C. Reaction was terminated by the addition of TFA and samples were analyzed via HPLC. Bactericidal effect of citrullinated LL-37 and hArg-LL-37 on (**C**) *E. coli*; (**D**) *P. aeruginosa;* and (**E**) *S. aureus* *Δaur* were determined using SYTO9 and PI staining, as described above. Citrullination of LL-37 and hArg-LL-37 was obtained by treatment of the native peptide and hArg-LL-37 with human PAD4 at 23.3 U/mg peptide. Mean ± SD *n* = 2. ns—non-significant; *** *p* < 0.001, **** *p* < 0.0001; (**F**) *E. coli* was mixed with either citrullinated or not citrullinated native LL-37 or hArg-LL-37 (50 μg/mL), followed by analysis of the inner membrane permeabilization by Sytox Green nucleic acid staining. Mean ± SD *n* = 2. ns—non-significant; ** *p* < 0.01; (**G**) The scheme presenting model of *E. coli* killing by PMA-derived NETs in the presence of LL-37 and hArg-LL-37; Antibacterial activity of LL-37 and hArg-LL-37 against *E. coli* was assessed in the PMA-induced NETs. Bacterial cells were exposed to NETs collected from PMA-activated neutrophils isolated from healthy donors supplemented with 25 μg/mL LL-37 or hArg-LL-37. After 2 h of incubation, suspensions were plated on agar plates for counting of bacteria. (**H**) The number of viable bacterial cells is expressed as a % of control with respect to the number of bacteria in the corresponding control samples (no stimulation). Mean ± SEM *n* = 3. **** *p* < 0.0001.

Citrullination significantly affects the microbicidal activity of LL-37 [11,13]. We hypothesized that substitution of arginine to homoarginine in LL-37 would protect the peptide against deimination catalyzed by PADs and thus preserve its antibacterial activity. To verify this hypothesis, we first showed that hArg-LL-37 was resistant to citrullination by PAD4, in contrast with LL-37. While PAD4 did not evidently modify hArg-LL-37, it modified LL-37 within 2 h, as illustrated by high-performance liquid chromatography analysis (Figure 4A,B). Next, we investigated if the bactericidal function of hArg-LL-37 was maintained in the presence of PAD4 by performing the LIVE/DEAD assay with *E. coli*, *P. aeruginosa*, and *Staphylococcus aureus*. To avoid degradation of LL-37 by staphylococcal proteases (Figure 3C) [18], we used the aureolysin-null *S. aureus* strain (*S. aureus* Δ*aur*). The bactericidal activity of hArg-LL-37 against all tested microorganisms was retained upon incubation with PAD4 (Figure 4C–E). This activity was apparently mediated by cell membrane permeabilization because disruption of the cytoplasmic membrane of *E. coli* was detected by the Sytox Green assay (Figure 4F). Finally, we determined whether hArg-LL-37 retains its bactericidal properties in a milieu that resembles in vivo conditions. For this purpose, we used NETs enriched in deiminases, proteases, and myeloperoxidase, which readily modify LL-37 (Figure 4G) [19,20,21]. NETs were supplemented with native LL-37 or hArg-LL-37 and then their bactericidal effects against *E. coli* were examined (Figure 4H). The intrinsic antibacterial activity of NETs was not enhanced by supplementation with LL-37, but was significantly increased by supplementation with hArg-LL-37 (Figure 4H). Collectively, these data show that hArg-LL-37 is not modified by PAD4 and maintains its activity, while citrullination abolishes the activity of native LL-37.

## 3. Discussion

Posttranslational modifications control the biological activities of AMPs. Wang reported that 1147 of 1755 natural AMPs annotated in the AMP database are modified [22]. Expression and activities of enzymes that catalyze these modifications are enhanced in inflammatory milieu and may affect the biological activities of both natural and synthetic AMPs. Experiments using synthetic LL-37 revealed that post-translational modifications including proteolysis [23], non-enzymatic conversion of lysine residues to homocitrulline (carbamylation) [24], and deimination of arginine residues (citrullination) [12,13,14] significantly influence the antimicrobial and immunomodulatory functions of LL-37. Peptidyl-deiminases can efficiently citrullinate LL-37 because all five arginine residues are substituted by citrulline [13,14]. This process seems to be physiologically relevant because citrullinated LL-37 was identified in vivo [11,25]. Here, we focused on citrullination of LL-37 and a strategy to prevent this modification. We used synthetic LL-37 in which arginine residues were substituted by citrullination-resistant homoarginine residues (hArg-LL-37). hArg-LL-37 retained its biological activity upon PAD4 treatment. We further demonstrated that microbicidal activity of hArg-LL-37 against Gram-negative and Gram-positive bacteria was maintained in the presence of purified PAD4. By contrast, citrullination of native LL-37 by PAD4 significantly impaired its bactericidal activity against *E. coli*, *P. aeruginosa*, and *S. aureus*, consistent with previously published data [11,13]. Resistance of hArg-LL-37 to citrullination was also examined in semi-in vivo conditions using NETs, where the activities of PADs are well documented [12,20,26,27]. As expected, hArg-LL-37, but not native LL-37, significantly increased the intrinsic bactericidal activity of NETs, which are enriched with PADs and mimic in vivo conditions.

We previously showed that native LL-37 is gradually degraded in NETs [28]. Moreover, the half-life of citrullinated LL-37 in NETs, sera from healthy donors, and synovial fluid from rheumatoid arthritis patients is significantly shorter than that of native LL-37 [12]. The enhanced proteolysis of citrullinated LL-37 contrasts with observations that homoarginine-modified proteins show increased resistance to proteolytic cleavage catalyzed by trypsin-like proteases [29]. The slower hydrolysis rate of homoarginine-containing peptides compared with their arginine-containing counterparts depends on the substrate-to-protease ratio [30]. Therefore, we examined the susceptibility of hArg-LL-37 to proteolysis. hArg-LL-37 was as stable as the native peptide in the presence of cathepsin G and *S. aureus*-derived proteases, but was slightly more prone to degradation catalyzed by neutrophil elastase. Nonetheless, it is clear that substitution of arginine residues with homoarginine renders LL-37 resistant to citrullination. Although the susceptibility of hArg-LL-37 to proteolysis by neutrophil elastase was greater than that of the native peptide, hArg-LL-37 increased the bactericidal activity of NETs, which contain a high concentration of elastase, emphasizing its therapeutic potential [19]. This unequivocally demonstrates that the therapeutic potential of hArg-LL-37 is superior to that of native LL-37.

Besides peptide stability, there are many obstacles to the development of peptide-based medicines for infections. Among them is the potential toxicity of a drug. To the best of our knowledge, no study has reported a toxic effect of systemic distribution of homoarginine [31]. Importantly, we demonstrated that toxicity of hArg-LL-37 do not differ from native LL-37 (Figure 2A). Moreover, we showed that hArg-LL-37 was as an efficient bactericidal/bacteriostatic agent as native LL-37 and that the two peptides had a similar ability to permeabilize the bacterial cell membrane. Finally, our findings revealed that hArg-LL-37 had similar immunomodulatory activities as native LL-37. These included the ability to neutralize endotoxin via aggregation with LPS to form amyloid-like structures and to prevent interactions between endotoxin and membrane-anchored molecules crucial for activation of macrophages such as CD14 and TLR4. The latter was manifested by inhibition of LPS- and LTA-induced release of TNF-α and IL-10 from human macrophages. Taken together, hArg-LL-37 is a good candidate to treat bacterial infections and inflammatory conditions due to its bactericidal and immunomodulatory activities, which are unaffected by PADs in inflammatory foci.

Recent epidemiological data show that the emergence of multidrug-resistant bacterial strains is a global health problem and therefore new drugs must be urgently developed. Current research is partly focused on peptide-based compounds. In 2013, Pergamum AB released a clinical study report of a randomized phase I/II trial of LL-37 for treatment of venous leg ulcers. The healing rate was significantly better in patients treated with LL-37 than in those treated with a placebo, and no safety or tolerability concerns were noted [32]. Nevertheless, LL-37 has not been approved for treatment, most likely due to its propensity to undergo citrullination, which abrogates its beneficial activities. We propose that hArg-LL-37, whose biological activities are unaffected by PADs, has great therapeutic potential to facilitate wound healing and resolve skin infections when applied topically. Considering the antimicrobial and wound-healing properties of LL-37, we speculate that topical application of cathelicidin peptides with homoarginine residues has great potential to treat skin infections. Remarkably, hArg-LL-37, which is not citrullinated by PADs, may act at inflammatory foci in vivo where the functions of native LL-37 are noticeably perturbed by citrullination. In conclusion, the presented findings provide a proof-of-concept for the designing of therapeutic peptides using homoarginine.

## 4. Materials and Methods

### 4.1. Peptide Synthesis and Purification

Native LL-37 and LL-37 with arginine residues substituted by homoarginine (hArg-LL-37) were synthesized using the method described previously [14]. The sequences of peptides are presented in Table 1. The lack of contamination of synthetic peptides with LPS was verified by using the Limulus amebocyte lysate test (Lonza).

### 4.2. Expression and Purification of Human Recombinant PAD4

Full length human PAD4 with N-terminal His-tag was expressed in *E. coli* BL21 (DE3) cells using the pET-16b vector. HisTag-PAD4 expression was induced with 0.5 mM isopropyl β-D-1-thiogalactopyranoside (IPTG) at OD_600nm_ = 0.6 and the culture was incubated at 25 °C for 16 h. After this time cells were harvested, lysed and soluble protein fraction was applied on Ni Sepharose Fast Flow resin (GE Healthcare, Life Science, USA). Fractions containing HisTag-PAD4 were further purified using combination of ion exchange and gel filtration chromatography on MonoQ 4.6/100PE and HiLoad Superdex S75 16/600 columns respectively (GE Healthcare, Life Science). Finally, HisTag-PAD4 was concentrated and frozen in storage buffer (50 mM Hepes pH 7.5 with 0.25 M NaCl, 3% glycerol). PAD4 activity was determined on N-α-benzoyl-L-arginine ethyl ester hydrochloride (BAEE) substrate with colorimetric assay [33].

### 4.3. Citrullination of LL-37 and hArg-LL-37 by Human PAD4

LL-37 and hArg-LL-37 (1 mg/mL) resuspended in PAD assay buffer (100 mM Tris-HCl, 5 mM CaCl_2_, and 5 mM DTT [pH 7.6]) were treated with recombinant human PAD4 (23 U/mg) for 2 h at 37 °C as described previously [14]. Citrullination was terminated by snap-freezing the samples.

### 4.4. HPLC Analysis of Native and Modified LL-37/hArg-LL-37

Samples containing 10 μg of LL-37/hArg-LL-37 were treated with 233 mU of recombinant PAD4-HisTag (in buffer containing 100 mm Tris, 5 mM DTT, pH 7.5) for 1 h at 37 °C. Reaction was terminated by addition of TFA (final concentration 0.1%) (Sigma) and samples were analyzed via HPLC. Separation was achieved on a reverse-phase column Aeris XB-C18, 3.6 μm, 4.6/150 (Phenomenex) connected to ÄKTAmicro (GE Healthcare). Peptides were eluted in the gradient of Phase A (0.1% TFA in water) and Phase B (80% acetonitrile, 0.08% TFA in water) and monitored at 215 nm.

### 4.5. Bacterial Strains and Cultures

*Escherichia coli* ATCC 25922 and *Pseudomonas aeruginosa* (clinical isolate) were inoculated from stocks into 20 mL LB Broth Lennox, while *Staphylococcus aureus* Δ*aur* (8325-4 Δ*aur*) into 20 mL TSB and grown overnight at 37 °C under constant rotation (180 rpm) to a mid-logarithmic growth phase. Then they were centrifuged at 5000× *g* for 5 min, washed in PBS, and resuspended in PBS to the desired OD (600 nm).

### 4.6. Minimum Inhibitory Concentration (MIC)

Antimicrobial activity of peptides was analyzed through determination of the MIC, defined as the minimal concentration that completely inhibits growth of a microorganism, according to Clinical and Laboratory Standards Institute (CLSI). Mueller-Hinton broth (MHB, Sigma-Aldrich) was used as the working medium for all bacterial strains. Briefly, inoculum was prepared from freshly grown cultures of bacteria being at their exponential phase of growth. Each well of 96-well plates containing 100 μL of serially diluted peptides in PBS at desired concentration was inoculated with 100 μL of 1 × 10^5^ (CFU) mL^−1^ of bacterial suspensions. Then, plates were incubated overnight at 37 °C and absorbance was read at 590 nm in a Flex Station 3 multi-mode microplate reader (Molecular Devices). An absorbance reading in wells without a peptide representing unrestrained bacterial growth was taken as positive control (100% bacterial survival). Readings in wells without bacteria were taken as negative controls. All measurements were run in triplicates.

### 4.7. Assessment of Bacterial Viability by Using the LIVE/DEAD BacLight KIT

Bacterial cells were collected at the end of the exponential/beginning of the stationary growth phase, washed and suspended at 2 × 10^7^ bacteria/mL. Next, bacteria were treated with LL-37 or hArg-LL-37 (2–200 μg/mL) for 2 h. In order to obtain a standard curve, five different proportions of live and dead bacteria were mixed prior to staining. After the incubation, the 100 μL of bacterial suspensions were transferred to a flat bottom black 96-well microtitration plate. Staining solution containing SYTO9 and PI (100 μL) prepared according to the manufacturer’s instructions and was then mixed with bacterial suspensions. Samples were incubated at room temperature in the dark for 15 min and fluorescence intensity was measured with FlexStation3 Multimode Microplate Reader (Molecular Device) using a 485 nm excitation filter (for both SYTO9 and PI) and a 530 nm (SYTO9 emission wavelength) and 630 nm (PI emission wavelength) emission filter. The data were analyzed by dividing the fluorescence intensity of the stained bacterial suspensions at green emission by the cell fluorescence intensity of red fluorescence. All samples were prepared in triplicates.

### 4.8. Sytox Green Uptake Analysis

To analyze inner membrane permeabilization, 5 × 10^7^ CFU/mL *E. coli* was incubated with LL-37 or hArg-LL-37 (50 μg/mL) for 30 min at 37 °C in LB. After incubation, bacteria were centrifuged at 1200× *g* for 10 min at 4 °C and washed with PBS. Next, bacteria were resuspended in PBS with 3 μM Sytox Green Nucleic Acid Stain (Thermo Fisher Scientific) and transferred to black 96-well plate. After 5 min incubation at room temperature, a FlexStation3 Multimode Microplate Reader (Molecular Device) was utilized to measure fluorescence intensity at two wavelengths (excitation at 504 nm and emission at 523 nm).

### 4.9. ThT Dye-Binding Assay

ThT (thioflavin T) dyes were used to detect amyloid fibrils. ThT preferentially binds to the β-sheet structures of amyloidogenic proteins and peptides. LPS (100 μg/mL) was incubated with LL-37 and hArg-LL-37 (100 μg/mL) or with buffer only (10 mM, pH 7.4 Tris) for 30 min at 37 °C. Then, 200 μL sample was incubated with ThT (final concentration 10 μM) for 15 min in the dark. ThT fluorescence was measured at an excitation/emission wavelength of 440/482 nm. The baseline (buffer) was subtracted from the sample signal. These measurements were performed on a FlexStation3 Multimode Microplate Reader (Molecular Devices).

### 4.10. Isolation and Culture of Primary Cells

Peripheral blood from de-identified human donors was purchased from the Red Cross (Krakow, Poland). Thus, the study did not require consent from the study subjects. Briefly, PBMCs were isolated from human blood using a lymphocyte separation medium density gradient as described before [34]. Cells from fraction highly enriched in monocytes (90% CD14 positive) were plated at 3 × 10^6^/well in 24-well plates (Sarstedt) in RPMI 1640 supplemented with 2 mM L-glutamine, 50 μg/mL gentamycin, and 10% autological human serum. After 24 h, nonadherent PBMCs were removed by washing with complete medium, and adherent cells were cultured in this medium for 7 days until they differentiated into macrophages (hMdMs, human monocyte-derived macrophages). Neutrophils were isolated by centrifugation over a density gradient from granulocyte-enriched fractions. Then the high-density fraction was harvested, and neutrophils were separated from erythrocytes after 30 min of incubation with 1% polyvinyl alcohol (POCH). Neutrophils were collected from the upper layer, and after centrifugation (280× *g*, 10 min), the residual erythrocytes were removed by using lysis in water. Neutrophils were resuspended in serum-free DMEM without phenol red (Gibco/ThermoFisher Scientific). The murine macrophage cell line RAW 264.7 obtained from the American Type Culture Collection was maintained in DMEM supplemented with 5% fetal bovine serum.

### 4.11. Cell Viability Test

The cell line of human immortalized keratinocytes (HaCaT) was obtained from American Type Culture Collection (ATCC, Manassas, VA, USA) and cultured in DMEM (Gibco, Life Technologies, Paisley, UK) supplemented with 10% heat-inactivated fetal bovine serum (FBS) and PEST (100 U/mL penicillin and 100 U/mL streptomycin) at 37 °C in humidified 5% CO_2_ atmosphere. Cells were passaged every 4–5 days. To assess the effect of peptides on the integrity of the plasma membrane, the LDH release assay was performed using the CytoTox96 nonradioactive cytotoxicity assay kit (Promega, Madison, WI, USA) according to the manufacturer’s instructions. Cytotoxicity was calculated with the formula: % cytotoxicity = 100 × (experimental LDH release/maximum LDH release), where maximum LDH release occurs after the addition of lysis solution (Triton X-100). Relative amounts of LDH release were measured (absorbance at 490 nm) using FlexStation 3 Multimode Microplate Reader (Molecular Device, Wokingham, UK). All assays were performed in duplicate.

### 4.12. Macrophages Stimulation

hMdMs at 3 × 10^5^/well in 24-well plates in RPMI 1640 supplemented with 10% human serum were incubated with 10 ng/mL LPS (from *E. coli* 0111:B4, Sigma-Aldrich), 10 μg/mL LTA and 10 μg/mL PGN in the presence of LL-37 or hArg-LL-37 for 20 h. Then the medium was harvested and the production of cytokines (TNF-α, IL-10) was estimated with an ELISA kit measured as per the manufacturer’s instructions (BD Biosciences).

### 4.13. Measurement of Cytokine Levels in Whole Blood

To measure cytokine release by human blood cells, fresh venous blood taken from healthy donors (collected in EDTA) was treated with 10 ng/mL *E. coli* (0111:B4)-derived LPS in the presence or absence of LL-37 and hArg-LL-37 (10 μg/mL). After 10 h of incubation at 37 °C/5% CO_2_, tubes were centrifuged for 10 min at 200× *g* without breaking and plasma was collected for cytokine analysis. The amount of TNF-α was measured using ELISA (BD Biosciences).

### 4.14. Binding of LPS to RAW 264.7 Cells

The effect of LL-37 and hArg-LL-37 on LPS binding to cells was determined as described previously [35]. RAW 264.7 cells (5 × 10^5^ cells) were incubated with 100 ng/mL of Alexa Fluor 488-conjugated LPS (055:B5, Molecular Probes) in DMEM supplemented with 5% FBS at 37 °C in the presence of 25 μg/mL LL-37 or hArg-LL-37. Cells were then washed twice with ice-cold PBS and LPS binding was analyzed by flow cytometry (FACSCalibur, BD Biosciences). The mean fluorescence intensity and percentage of cells labeled with Alexa Fluor 488-conjugated LPS was measured in each group.

### 4.15. Degradation of LL-37 and hArg-LL-37 by Human Proteases

LL-37 and hArg-LL-37 (5 μg) in PBS were incubated alone or in the presence of human proteases: neutrophil elastase (0.05 μg) and cathepsin G (0.5 μg) or bacterial proteases V8 (0.05 μg), aureolysin (0.05 μg) in total volume 25 μL at 37 °C for 1 h and 18 h. After incubation samples were immediately frozen at −80 °C. Samples were separated on SDS-PAGE under denaturing condition in 4–16% gels. After separation, the gels underwent colloid Coomassie staining.

### 4.16. NETs-Mediated Bacterial Killing in the Presence of LL-37 or hArg-LL-37

Human neutrophils were seeded at 2 × 10^6^/well in 0.01 mg/mL poly-L-lysine (Sigma-Aldrich)-coated 24-well plates and centrifuged (200× *g*, 5 min) to allow cells to adhere to the plates. Then, neutrophils were stimulated with 25 nM phorbol ester (PMA; Sigma-Aldrich) for 4 h at 37 °C. LL-37 or hArg-LL-37 was added (final concentration 25 μg/mL) for 30 min prior to end of stimulation. Extruded NETs were collected and incubated at 37 °C with *E. coli* at a MOI of 1:5 (based on the number of neutrophils from which the NETs were collected). After 2 h, bacterial survival was estimated by plating dilutions on agar plates and counting colonies of bacteria. The number of viable bacterial cells is expressed as a percentage of control, where bacteria were incubated in serum-free DMEM without neutrophils.

### 4.17. Statistical Analysis

Data were analyzed by GraphPad Prism version 6.0 (GraphPad software). Parametric tests unpaired student’s *t* test or ANOVA were used. A *p* value of <0.05 was used for statistical significance.

## Figures and Tables

**Table 1:** Sequences of LL-37 used in the study.

Peptide	Sequence
LL-37	LLGDFFRKSKEKIGKEFKRIVQRIKDFLRNLVPRTES
hArg-LL-37	LLGDFF(hR)KSKEKIGKEFK(hR)IVQ(hR)IKDFL(hR)NLVP(hR)TES

hR: homoarginine (hArg).

## Abbreviations

NETs	neutrophil extracellular traps
PAD	peptidyl-arginine deiminases
AMPs	antimicrobial peptides
LTA	lipoteichoic acid
PGN	peptidoglycan
IPTG	isopropyl β-D-1-thiogalactopyranoside
BAEE	N-α-benzoyl-L-arginine ethyl ester hydrochloride
PBS	phosphate buffered saline
OD	optical density
MIC	minimal inhibitory concentration
CFU	colony forming units
MHB	Mueller-Hinton broth
PI	propidium iodide
LB	Lennox broth
ThT	thioflavin T
PBMCs	peripheral blood mononuclear cell
hMdMs	human monocyte-derived macrophages
DMEM	Dulbecco’s Modified Eagle’s Medium
FBS	fetal bovine serum
LDH	lactate dehydrogenase
RPMI	Roswell Park Memorial Institute Medium
EDTA	ethylenediaminetetraacetic acid
ELISA	enzyme-linked immunosorbent assay
MOI	multiplicity of infection

## References

[1] Boman H.G. (1991). Antibacterial peptides: Key components needed in immunity. Cell.

[2] Hancock R.E., Haney E.F., Gill E.E. (2016). The immunology of host defence peptides: Beyond antimicrobial activity. Nat. Rev. Immunol..

[3] Agerberth B., Gunne H., Odeberg J., Kogner P., Boman H.G., Gudmundsson G.H. (1995). FALL-39, a putative human peptide antibiotic, is cysteine-free and expressed in bone marrow and testis. Proc. Natl. Acad. Sci. USA.

[4] Sørensen O.E., Follin P., Johnsen A.H., Calafat J., Tjabringa G.S., Hiemstra P.S., Borregaard N. (2001). Human cathelicidin, hCAP-18, is processed to the antimicrobial peptide LL-37 by extracellular cleavage with proteinase 3. Blood.

[5] Yamasaki K., Schauber J., Coda A., Lin H., Dorschner R.A., Schechter N.M., Bonnart C., Descargues P., Hovnanian A., Gallo R.L. (2006). Kallikrein-Mediated proteolysis regulates the antimicrobial effects of cathelicidins in skin. FASEB J..

[6] Oren Z., Lerman J.C., Gudmundsson G.H., Agerberth B., Shai Y. (1999). Structure and organization of the human antimicrobial peptide LL-37 in phospholipid membranes: Relevance to the molecular basis for its non-cell-selective activity. Biochem. J..

[7] Travis S.M., Anderson N.N., Forsyth W.R., Espiritu C., Conway B.D., Greenberg E.P., McCray P.B., Lehrer R.I., Welsh M.J., Tack B.F. (2000). Bactericidal activity of mammalian cathelicidin-derived peptides. Infect. Immun..

[8] Nagaoka I., Hirota S., Niyonsaba F., Hirata M., Adachi Y., Tamura H., Tanaka S., Heumann D. (2002). Augmentation of the lipopolysaccharide-neutralizing activities of human cathelicidin CAP18/LL-37-derived antimicrobial peptides by replacement with hydrophobic and cationic amino acid residues. Clin. Diagn. Lab. Immunol..

[9] Cirioni O., Giacometti A., Ghiselli R., Bergnach C., Orlando F., Silvestri C., Mocchegiani F., Licci A., Skerlavaj B., Rocchi M. (2006). LL-37 protects rats against lethal sepsis caused by gram-negative bacteria. Antimicrob. Agents Chemother..

[10] Vossenaar E.R., Zendman A.J., van Venrooij W.J., Pruijn G.J. (2003). PAD, a growing family of citrullinating enzymes: Genes, features and involvement in disease. Bioessays.

[11] Al-Adwani S., Wallin C., Balhuizen M.D., Veldhuizen E.J.A., Coorens M., Landreh M., Végvári Á., Smith M.E., Qvarfordt I., Lindén A. (2020). Studies on citrullinated LL-37: Detection in human airways, antibacterial effects and biophysical properties. Sci. Rep..

[12] Wong A., Bryzek D., Dobosz E., Scavenius C., Svoboda P., Rapala-Kozik M., Lesner A., Frydrych I., Enghild J., Mydel P. (2018). A novel biological role for peptidyl-arginine deiminases: Citrullination of Cathelicidin LL-37 controls the immunostimulatory potential of cell-free DNA. J. Immunol..

[13] Kilsgård O., Andersson P., Malmsten M., Nordin S.L., Linge H.M., Eliasson M., Sörenson E., Erjefält J.S., Bylund J., Olin A.I. (2012). Peptidylarginine deiminases present in the airways during tobacco smoking and inflammation can citrullinate the host defense peptide LL-37, resulting in altered activities. Am. J. Respir. Cell Mol. Biol..

[14] Koziel J., Bryzek D., Sroka A., Maresz K., Glowczyk I., Bielecka E., Kantyka T., Pyrć K., Svoboda P., Pohl J. (2014). Citrullination alters immunomodulatory function of LL-37 essential for prevention of endotoxin-induced sepsis. J. Immunol..

[15] Méchin M.C., Sebbag M., Arnaud J., Nachat R., Foulquier C., Adoue V., Coudane F., Duplan H., Schmitt A.M., Chavanas S. (2007). Update on peptidylarginine deiminases and deimination in skin physiology and severe human diseases. Int. J. Cosmet. Sci..

[16] Foulquier C., Sebbag M., Clavel C., Chapuy-Regaud S., Al Badine R., Méchin M.C., Vincent C., Nachat R., Yamada M., Takahara H. (2007). Peptidyl arginine deiminase type 2 (PAD-2) and PAD-4 but not PAD-1, PAD-3, and PAD-6 are expressed in rheumatoid arthritis synovium in close association with tissue inflammation. Arthritis Rheum..

[17] Marshall N.C., Finlay B.B., Overall C.M. (2017). Sharpening host defenses during infection: Proteases cut to the chase. Mol. Cell. Proteom..

[18] Sieprawska-Lupa M., Mydel P., Krawczyk K., Wójcik K., Puklo M., Lupa B., Suder P., Silberring J., Reed M., Pohl J. (2004). Degradation of human antimicrobial peptide LL-37 by Staphylococcus aureus-derived proteinases. Antimicrob. Agents Chemother..

[19] Brinkmann V., Reichard U., Goosmann C., Fauler B., Uhlemann Y., Weiss D.S., Weinrauch Y., Zychlinsky A. (2004). Neutrophil extracellular traps kill bacteria. Science.

[20] Wang Y., Li M., Stadler S., Correll S., Li P., Wang D., Hayama R., Leonelli L., Han H., Grigoryev S.A. (2009). Histone hypercitrullination mediates chromatin decondensation and neutrophil extracellular trap formation. J. Cell Biol..

[21] Cooper P.R., Palmer L.J., Chapple I.L. (2013). Neutrophil extracellular traps as a new paradigm in innate immunity: Friend or foe?. Periodontology 2000.

[22] Wang G. (2014). Human antimicrobial peptides and proteins. Pharmaceuticals.

[23] McCrudden M.T.C., McLean D.T.F., Zhou M., Shaw J., Linden G.J., Irwin C.R., Lundy F.T. (2014). The host defence peptide LL-37 is susceptible to proteolytic degradation by wound fluid isolated from foot ulcers of diabetic patients. Int. J. Pept. Res. Ther..

[24] Koro C., Hellvard A., Delaleu N., Binder V., Scavenius C., Bergum B., Główczyk I., Roberts H.M., Chapple I.L., Grant M.M. (2016). Carbamylated LL-37 as a modulator of the immune response. Innate Immun..

[25] Lande R., Palazzo R., Hammel P., Pietraforte I., Surbeck I., Gilliet M., Chizzolini C., Frasca L. (2020). Generation of monoclonal antibodies specific for native LL37 and citrullinated LL37 that discriminate the two LL37 forms in the skin and circulation of cutaneous/systemic lupus erythematosus and rheumatoid arthritis patients. Antibodies.

[26] Rohrbach A.S., Slade D.J., Thompson P.R., Mowen K.A. (2012). Activation of PAD4 in NET formation. Front. Immunol..

[27] Lewis H.D., Liddle J., Coote J.E., Atkinson S.J., Barker M.D., Bax B.D., Bicker K.L., Bingham R.P., Campbell M., Chen Y.H. (2015). Inhibition of PAD4 activity is sufficient to disrupt mouse and human NET formation. Nat. Chem. Biol..

[28] Bryzek D., Ciaston I., Dobosz E., Gasiorek A., Makarska A., Sarna M., Eick S., Puklo M., Lech M., Potempa B. (2019). Triggering NETosis via protease-activated receptor (PAR)-2 signaling as a mechanism of hijacking neutrophils function for pathogen benefits. PLoS Pathog..

[29] Izdebski J., Kunce D., Schiller P.W., Chung N.N., Gers T., Zelman M., Grabek M. (2007). Synthesis and biological activity of homoarginine-containing opioid peptides. J. Pept. Sci..

[30] Chowdhury S.M., Munske G.R., Yang J., Zhukova D., Nguyen H., Bruce J.E. (2014). Solid-Phase N-terminal peptide enrichment study by optimizing trypsin proteolysis on homoarginine-modified proteins by mass spectrometry. Rapid Commun. Mass Spectrom..

[31] Rodionov R.N., Begmatov H., Jarzebska N., Patel K., Mills M.T., Ghani Z., Khakshour D., Tamboli P., Patel M.N., Abdalla M. (2019). Homoarginine supplementation prevents left ventricular dilatation and preserves systolic function in a model of coronary artery disease. J. Am. Heart Assoc..

[32] Grönberg A., Mahlapuu M., Ståhle M., Whately-Smith C., Rollman O. (2014). Treatment with LL-37 is safe and effective in enhancing healing of hard-to-heal venous leg ulcers: A randomized, placebo-controlled clinical trial. Wound Repair Regen..

[33] Liao Y.F., Hsieh H.C., Liu G.Y., Hung H.C. (2005). A continuous spectrophotometric assay method for peptidylarginine deiminase type 4 activity. Anal. Biochem..

[34] Koziel J., Maciag-Gudowska A., Mikolajczyk T., Bzowska M., Sturdevant D.E., Whitney A.R., Shaw L.N., DeLeo F.R., Potempa J. (2009). Phagocytosis of *Staphylococcus aureus* by macrophages exerts cytoprotective effects manifested by the upregulation of antiapoptotic factors. PLoS ONE.

[35] Nagaoka I., Hirota S., Niyonsaba F., Hirata M., Adachi Y., Tamura H., Heumann D. (2001). Cathelicidin family of antibacterial peptides CAP18 and CAP11 inhibit the expression of TNF-alpha by blocking the binding of LPS to CD14(+) cells. J. Immunol..

